# Initial self-blame predicts eating disorder remission after 9 years

**DOI:** 10.1186/s40337-021-00435-3

**Published:** 2021-07-07

**Authors:** Suzanne Petersson, Andreas Birgegård, Lars Brudin, Emma Forsén Mantilla, Elin Monell, David Clinton, Caroline Björck

**Affiliations:** 1 Department of Rehabilitation, Kalmar Regional Council, Hus 13, plan 7, 391 85, Länssjukhuset, Kalmar, Sweden; 2 Department for Medicine and Optometry, Linnaeus University, Kalmar, Sweden; 3 Department of Medical Epidemiology and Biostatistics, Karolinska Institutet, Stockholm, Sweden; 4 Department of Clinical Physiology, Region Kalmar County, Kalmar, Sweden; 5 Department of Medical and Health Sciences, Linköping University, Linköping, Sweden; 6 Centre for Psychiatry Research, Stockholm Health Care Services, Karolinska Institutet, Stockholm, Sweden; 7 Department of Clinical Neuroscience, Karolinska Institutet, Stockholm, Sweden; 8 Institute for Eating Disorders, Villa Sult, Oslo, Norway; 9 Department of Neurobiology, Care Sciences and Society (NVS), Karolinska Institutet, Stockholm, Sweden

**Keywords:** Eating disorders, Outcome, Prediction, Self-image

## Abstract

**Background:**

Research into predictors of outcome in eating disorders (ED) has shown conflicting results, with few studies of long-term predictors and the possible importance of psychological variables that may act as risk- and maintenance factors.

**Aim:**

To identify baseline predictors of ED remission nine years after initial clinical assessment using self-report measures of ED psychopathology, psychiatric symptoms, and self-image in a sample of adult ED patients (*N* = 104) treated at specialist units in Stockholm, Sweden. Sixty patients participated in the follow-up, of whom 41 patients (68%) had achieved remission.

**Results:**

Results suggested that the only significant predictor of diagnostic remission after nine years was initial levels of self-blame.

**Conclusion:**

In order to ensure long-term recovery in ED it may be important for clinicians to widen their therapeutic repertoire and utilise techniques that reduce self-blame and increase self-compassion.

**Plain English summary:**

It is difficult to predict how an eating disorder will develop, and research has found varying factors that affect the outcome of the condition. Recovery rates vary from nearly nil to over 90%. This variation could be explained by different research factors, but are more likely due to varying definitions of ‘recovery’, with less stringent definitions yielding high recovery rates and more stringent definitions yielding lower rates. The present study investigated whether the severity of eating disorder symptoms and other psychiatric symptoms could predict recovery nine years from first admission to specialised eating disorder care. Sixty patients at three eating disorder treatment units participated, and their scores on self-report measures of symptoms were used as predictor variables. Forty-one participants had no eating disorder diagnosis at nine-year follow-up. Most participants with binge-eating disorder had recovered, while the poorest outcome was found for anorexia nervosa with slightly over half of patients recovered after nine years. The only predictor for the nine-year outcome was a higher initial rating of self-blame, measured with the Structural Analysis of the Social Behavior. It was concluded that it may be important for clinicians to detect and address self-blame early in the treatment of eating disorders in order to enhance the possibility of recovery. Treatment should focus on reducing self-blame and increasing self-acceptance.

## Introduction

Research into predictors of outcome in eating disorders (ED) has shown conflicting results [1]. This may be due to length of follow-up, definitions of outcome, characteristics of the samples studied, and the assessment methods used. In a meta-analysis of 126 studies Vall and Wade [1] found that the most robust predictor outcome at end of treatment and follow-up was symptom change during early treatment. Other important variables included illness duration, weight and shape concern, general psychopathology, ED pathology, and self-esteem. However, among the 126 studies examined by Vall and Wade [1], follow-ups after more than two years were rare, few instruments assessed psychological variables that may act as both risk- and maintenance factors, while definitions of outcome and remission varied considerably.

Some studies that have investigated initial predictors of long-term outcome have found that poor long-term outcome is associated with poor social relationships prior to onset of illness, as well as extreme and compulsive drive to exercise [2]; high weight- and shape concern [3]; high scores on the Eating Disorder Inventory [4]; drive for thinness [5]; maturity fears [6]; obsessive-compulsive symptoms [7]; and depression [8]. Initial negative self-image (particularly self-hate) seems to be associated with a less favorable outcome after three years [9, 10]. Moreover, lower scores on self-blame and higher scores on self-love seem to predict positive 12-month outcome [11]. In these studies, self-image was measured using the Structural Analysis of Social Behavior (SASB) [12, 13] model, which has demonstrated associations with a range of ED longitudinal features and outcomes, including suicidality and dropout from treatment [14, 15]; yet thus far the importance of self-image for long-term outcome remains unexplored. SASB stems from interpersonal theory, where self-image is a personality trait-level construct, and is expected to be relatively stable over time [12]. Given the central role that self-image seems to have as a maintenance factor in EDs, its potential value as a predictor of long-term outcome deserves attention.

Recovery rates for ED vary from nearly nil to 92% [6, 16]. Such dramatic variation between studies may be due to factors such as the variables investigated, follow-up times, sample makeup, and attrition. Discrepancies in recovery rates may also, importantly, be due to varying definitions of the outcome variable “recovery” [17–20].

One definition of recovery is that criteria for diagnostic remission have been met for a longer period than required according to diagnostic manuals. However, there is little agreement on the duration of the asymptomatic period required, which has varied between one month and five years in different studies, although three, six, or twelve months is most common [19]. Established diagnostic manuals are of little help. DSM-IV [21] had no recommended time span for defining remission, and although DSM-5 takes steps in the right direction it remains vague, defining full remission as “none of the criteria have been met for a sustained period of time” [21]. In a review of ED recovery Bardone-Cone and colleagues [19] suggested that the definition should include absence of diagnostic criteria for any ED plus scores on psychological ED measures that are indistinguishable from healthy controls but significantly different from people with EDs [19].

In the present study we aimed to explore the potential of self-image, as well as the previously researched outcome predictors of psychiatric comorbidity and severity of ED psychopathology, as baseline predictors of ED remission nine years after first admission to specialist ED care. Since previous results regarding the long-term impact of baseline psychopathology and comorbidity are contradictory, and studies focusing self-image and long-term outcome in EDs are lacking, we did not stipulate any hypotheses in relation to these predictors.

## Method

### Participants

A consecutive series of adult ED patients (over 18 years of age) entering treatment at either of three publicly funded specialist units in Stockholm, Sweden, between August 2001 and July 2002 was recruited. Initially, a total of 118 patients agreed to participate; 12 declined participation before baseline and initial data for two participants were missing, which meant that 104 participants entered the study. Sixty of these (58%), all women, completed the same assessments nine years later with complete data and constitute the participants in the present study. Reasons for missing data at follow-up included dropping out from treatment and non-participation in assessment, as well as failure to obtain data on all measures. There were no significant differences between drop outs and remaining participants regarding baseline diagnoses (*х*^2^ = 4.86, α = .05), age (drop outs *M* = 23.4, *SD* = 6.1), BMI (drop outs *M* = 21.0, *SD* = 6.4, range 12.9–38.8), or baseline predictor variable scores (see Table 1 for participants’ age and BMI). Participating treatment units offered a wide variety of interventions, such as individual, family or group therapies, nutritional treatment, psychopharmacological medication, and expressive forms of treatment, such as art therapy. Although treatment was individually tailored, general foci were restoration of normal eating patterns, problems with self-image, interpersonal relationships, and affect-related difficulties. Data from the present database have been used previously to examine dietary habits [22].

**Table 1:** Means and standard deviations (*SD*) for baseline variables per outcome group: recovered (= No eating disorder) versus still suffering from eating disorder after 9 years, logistic regression for baseline differences as well as Cohen’s *d* effect sizes for pre-post mean differences. Significant *p*-value in **bold**

		Univariate		
Variable	Mean (***SD***)	OR (95% CI)	***p***	Effect sizes^**2**^
**Age (years)**				
**Eating Disorder**	26.1 (7.5)	1.00		
**No Eating Disorder**	24.8 (5.4)	0.97 (0.88–1.06)	0.449	
**Body Mass Index (kg/m**^**2**^**)**				
**Eating Disorder**	20.9 (5.3)	1.00		
**No Eating Disorder**	23.3 (8.0)	1.06 (0.96–1.16)	0.252	
**EDI-DT**				
**Eating Disorder**	14.9 (5.0)	1.00		0.59
**No Eating Disorder**	15.0 (4.5)	1.0 (0.9–1.1)	0.950	1.61
**EDI-BULIMIA**				
**Eating Disorder**	5.3 (4.7)	1.00		0.31
**No Eating Disorder**	7.2 (6.1)	1.06 (0.95–1.19)	0.254	1.38
**EDI-BD**				
**Eating Disorder**	18.5 (7.3)	1.00		0.31
**No Eating Disorder**	18.9 (7.5)	1.01 (0.93–1.09)	0.839	1.1
**SCL 90 index**				
**Eating Disorder**	1.69 (0.75)	1.00		0.49
**No Eating Disorder**	1.68 (0.62)	0.98 (0.40–2.42)	0.972	1.52
**SASB C2**				
**Eating Disorder**	18.9 (15.5)	1.00		1.24
**No Eating Disorder**	23.2 (17.4)	1.02 (0.98–1.05)	0.364	1.44
**SASB C3**				
**Eating Disorder**	25.4 (15.2)	1.00		0.83
**No Eating Disorder**	30.1 (19.0)	1.02 (0.98–1.05)	0.351	1.3
**SASB C4**				
**Eating Disorder**	38.1 (22.1)	1.00		0.44
**No Eating Disorder**	44.6 (17.2)	5.2 (0.4–75.2)	0.223	0.71
**SASB C6**				
**Eating Disorder**	73.4 (20.9)	1.00		0.96
**No Eating Disorder**	59.6 (23.5)	0.97 (0.95–1.00)	**0.044**	1.35
**SASB C7**				
**Eating Disorder**	62.8 (21.7)	1.00		0.92
**No Eating Disorder**	51.5 (23.1)	0.98 (0.95–1.00)	*0.088*	1.3

Abbreviations: *EDI* Eating Disorder Inventory-2, *DT* Drive for Thinness, *BD* Body Dissatisfaction, *SCL* Symptom Check List-90, *SASB* Structural Analysis of Social Behaviour. *C2* Self-affirmation, *C3* Self-love, *C4* Self-protection, *C6* Self-blame, and *C7* Self-hate

^2^Pre-post effect sizes calculated with Cohen’s *d*

### Measures

A battery of interviews and self-report measures was used to assess ED, general psychopathology, and relevant psychological variables. The present study utilised a subset of self-report measures.

The Rating of Anorexia and Bulimia (RAB) [23], a semi-structured interview, was used for assessment of DSM-IV ED [21) diagnoses at admission and follow-up. The RAB-R has been validated in a clinical sample of Swedish eating disorder patients (*n* = 71) together with a sample of healthy female controls (*n* = 31) and the test has shown satisfactory psychometric properties regarding internal consistency, inter-rater and test-retest reliability It correlated well with related measures, and discriminated between patients and normal controls.

The Eating Disorders Inventory-2 (EDI-2) [4], a self-report questionnaire with 91 items rated on a 6-point scale with eleven subscales measuring ED symptoms and related psychological variables. Symptom subscales measuring Drive for thinness (Cronbach’s alpha in this study was .81), Bulimia (*α* = .87), and Body Dissatisfaction (*α* = .91) were used in the study. The Swedish version of the EDI-2 has been validated in three female samples: patients with ED (*n* = 978); psychiatric outpatients (*n* = 106); and healthy controls (*n* = 602). Ages ranged from 18 to 50 years. Subscales showed high internal consistency and good test-retest reliability and the instrument and its subscales discriminated well between ED patients and healthy controls as well as between different ED diagnoses [24].

The Structural Analysis of Social Behavior (SASB, 3rd surface, self-image) [12, 13] utilises 36 self-referential statements that are rated 0–100 with 10-point increments to assess eight aspects (clusters) of self-image: C1) Self-emancipation; C2) Self-affirmation; C3) Active self-love; C4) Self-protection; C5) Self-control; C6) Self-blame; C7) Self-hate; and C8) Self-neglect. The Swedish version of the SASB was validated in a sample with healthy controls (*n* = 52, 54% women) and patients with different psychiatric diagnoses (*n* = 173, 42% women). The instrument has shown satisfactory psychometric properties regarding internal consistency, and test-retest reliability [25]. Internal consistency and correspondence between U.S. and Swedish versions are good [13, 25, 26]. The SASB has shown to discriminate well between clinical and normal samples [13, 25–28]. Measures of Cronbach’s alpha in the present sample were good for: Self-affirmation *α* = .76; Active Self-love *α* = .77; Self-protection *α* = .82; Self-blame *α* = .82; and Self-hate *α* = .81; but not for Self-neglect *α* = .51; Self-emancipation *α* = .45; or Self-control *α* = .41. Since lower internal consistency (i.e. < .70) for the latter three clusters has also been found in previous studies of Swedish ED patients [29], these clusters were removed from analyses.

The Symptom Check List-90 (SCL-90) [30, 31] was used to measure self-reported psychiatric symptoms. It consists of 90 items rated on a 5-point scale, with 10 subscales: Somatization; Obsession-compulsion; Interpersonal Sensitivity; Depression; Anxiety; Hostility; Phobic Anxiety; Paranoid Ideation; Psychoticism; and Additional Scales. The Swedish version has been validated in healthy controls (*n* = 1016, 73% women, age range = 18–63 years) and samples with different psychiatric diagnoses (*n* = 1782, 56% women, age range = 19–73 years). For this study the total sum index of all subscales was used to measure general psychiatric health (α=.97).

### Procedure

Administration of intake measures took place prior to treatment or within two to four weeks after commencing treatment. Two trained and experienced ED specialists (one specialist nurse and one psychologist and psychotherapist) conducted baseline and follow-up interviews. At 9-year follow-up, participants were contacted by letter or phone if they were no longer in treatment (true for all but five participants), and an appointment for a follow-up interview at the unit was made. Definition of remission from ED was operationalized as not fulfilling diagnostic criteria for any DSM-IV ED. Self-report measures were mailed to participants and they were asked to return them prior to the interview. If participants were unable to attend personally, telephone interviews were conducted.

### Data analysis

Statistical analyses were performed using STATISTICA 12.0. Logistic regression was used to examine differences between baseline variables and ED remission status after nine years, followed by multiple logistic regression when *p* < 0.1. Association between 9-year remission status and baseline ED diagnosis was examined using *х*^2^.

## Results

Table 1 presents measures of central tendency and dispersion for baseline variables in relation to remission or non-remission after nine years, as well as significance of between-group comparisons using logistic regression.^1^ There were no significant between-group differences on any baseline variables except for SASB Self-blame (*p* = .044); patients who were later in remission showed significantly lower levels of self-blame at initial presentation compared to patients who later were not in remission and for SASB Self-hate (*p* = 0.088); patients who were later in remission showed significantly lower levels of self-hate at initial presentation compared to patients who were later not in remission.

For those variables with *p* < .10, multiple logistic regression was used to examine the relationship between initial self-assessment measures and ED remission at nine-year follow-up. SASB Self-blame was the only significant predictor of remission: odds ratio = .97 (CI .95–1.0), *p* = .041. Higher baseline levels of self-blame were associated with lower likelihood of remission nine years later. When comparing initial ED diagnoses at baseline for differences in remission status after nine years using *х*^2^, no significant results were found.

An analysis was performed on recovery and possible diagnostic crossover from baseline to nine-year follow-up for those who still had an ED diagnosis at follow-up (Table 2). Of the 60 patients who participated at follow-up 41 (68%) were recovered. Recovery was not significantly different as a function of baseline diagnosis. When diagnostic crossover was examined for those patients who were not in remission, the most frequent crossovers were from Anorexia Nervosa (AN) to Other Specified Feeding and Eating Disorders (OSFED), from Bulimia Nervosa (BN) to OSFED, and from OSFED to BN. Forty-four percent of patients with initial AN still had an ED diagnosis at follow-up; about half of these patients had an OSFED diagnosis.

**Table 2:** Distribution of baseline diagnoses for those with and without an ED diagnosis at nine-year follow-up, *N* and percent in relation to status at follow-up

Baseline Diagnosis	Baseline *N*	No ED at follow-up *N* (%)	ED at follow-up *N* (%)
AN	16	9 (56.2%)	7 (43.8%)
BN	17	13 (76.5%)	4 (23.5%)
BED	10	8 (80%)	2 (20%)
OSFED	17	11 (64.7%)	6 (35.3%)
Total	60	41 (68.3%)	19 (31.7%)

Abbreviations: *AN* Anorexia Nervosa, *BN* Bulimia Nervosa, *BED* Binge Eating Disorder, *OSFED* Other Specified Feeding and Eating Disorders

## Discussion

The present study explored whether initial levels of ED psychopathology, psychiatric symptoms, and self-image could predict ED diagnostic remission after nine years, and found that the only significant predictor of remission was initial levels of self-blame. In particular, lower self-blame at initial assessment was associated with higher likelihood of remission nine years later.

AN had the lowest remission rate (56%), while Binge Eating Disorder (BED) had the highest (80%), which is also in line with previous findings [32]. As regards the EDI-2 and SCL-90, which are frequently used in ED research, previous findings have varied regarding the ability of these instruments to predict outcome. Ametller and colleagues [33] found that high scores on the Bulimia subscale of the EDI-2 predicted whether young patients with AN were still in inpatient treatment after six months, while Quadflieg and Fichter [5] found that EDI-2 Drive for Thinness could predict outcome more than eleven years after admission in BN.

High scores on the SCL-90 has been shown to predict ED diagnosis at follow-up in some studies [34, 35], but not others [36]. In the present study the lack of association between the EDI-2 and the SCL-90 on the one hand, and outcome on the other hand, may be due to sample differences or the salience of scantly researched psychological variables (such as self-blame) on long-term outcome. Another possibility for the lack of significant findings in relation to the EDI-2 and the SCL-90 in the present study may be because these instruments measure state-dependent aspects of ED, which fluctuate over time. They may therefore be more relevant for predicting short- and medium-term outcome, whereas the SASB may be more germane for measuring important trait-level personality-based changes over the long-term [12]. Length of follow-up in comparable previous studies was considerably shorter than the present study, with the exception of Quadflieg and Fichter [5] who did not use the SASB.

The relationship between ED and negative self-evaluation as measured by the SASB is well documented [37]. SASB Self-blame, in particular, contains items about accusing and punishing oneself, such as “I accuse and blame myself until I feel guilty, bad and ashamed” and “I make myself do and be things which are known not to be right for me. I fool myself”. Such items may be particularly relevant to ED.

It has been argued that ED symptoms may function to regulate negative emotions and aversive thoughts [38]. ED symptoms may act to improve self-perceived flaws in appearance or achievement [39–41]. Self-blame may play a central role in such a functional system by reinforcing negative attitudes toward self and body, and thereby stimulating increased ED behaviours (cf. [42]). Forsén Mantilla and co-workers [28] found that high scores on SASB Self-blame and low scores on SASB Self-affirmation were associated with ED symptoms.

Viewed in a clinical context, focusing on self-criticism may be essential for achieving long-term recovery from ED. Indeed, initial negative self-image (i.e. high levels of SASB Self-blame and Self-hate) can be reduced to normal levels after ED treatment [43]. The road to long-term recovery in ED may, therefore, not just require symptom-focused interventions, but also therapeutic techniques that increase self-compassion, the opposite of self-blame. It may be important for therapists to confront self-critical thinking and help patients to understand, tolerate, and accept perceived flaws and weaknesses. One way of working actively with self-blame is Compassion-focused Therapy (CFT) where patients are encouraged to develop non-judgemental, compassionate self-correction in relation to one’s mistakes and shortcomings, as well as reduce self-criticism and self-blame [44, 45]. Thus, it may be essential to integrate components of CFT with standard symptom-focused interventions to achieve optimal outcome [46]. Future research could address the question of whether optimal long-term outcome in ED patients generally or in specific subgroups is mediated by interventions that reduce self-blame.

### Limitations

Strengths of the study were its longitudinal design with a nine-year follow-up and the use of well validated instruments that assessed important psychological variables relevant to ED as well as indices of ED and general psychopathology. Another strength was the naturalistic sample, comprising patients with a variety of ED diagnoses, ages, and a wide range of psychiatric symptoms as measured by the SCL-90, which increases representativeness and generalisability in relation to adult ED patients as they present in the community.

Nevertheless, there were also a number of limitations. The sample size was relatively small for this type of investigation, which reduces power and thus the generalizability. Self-report data was used, entailing risks of response bias. However, using well established, valid, and reliable measures ensured that the study variables were assessed in the same way. Data on number of previous treatment episodes, treatment duration, and current treatment could not be collected, which meant it was not possible to use such variables as covariates. The relevance of the outcome measure (diagnostic remission) can be criticised for being crude. As Bardone-Cone and colleagues [19] have pointed out, diagnostic remission can be considered to be a necessary but insufficient definition of recovery. Future research may therefore do well to utilise a more multifaceted measure of outcome.

## Conclusion

These results confirm previous findings of the predictive capacity of the SASB self-image, and underscore the centrality of self-blame, self-criticism, or in SASB parlance *negative self-control*, for ED symptomatology. These findings may suggest that when such tendencies are detected in the clinic, therapeutic interventions should focus on reducing self-blame and fostering self-acceptance.

### Clinical implications

Despite a plethora of research, ED remain difficult to treat and treatment results are often disheartening for patients, families and therapists. By attuning to tendencies toward self-blame early in treatment and fostering the development of a healthier self-image it may be possible to help patients move in a positive direction.

### Clinical implications

Despite the amount of research in the field, ED are difficult to treat and results are often disheartening. By paying attention to patient tendencies to self-blame early in treatment and contributing to the development of a healthier self-image the development of the condition could progress in a positive direction.

## Data Availability

The datasets generated during and/or analysed during the current study are not publicly available due to patient confidentiality.

## Abbreviations

AN: Anorexia Nervosa
BMI: Body Mass Index
BED: Binge Eating Disorder
BN: Bulimia Nervosa
CFT: Compassion-focused Therapy
DSM: Diagnostic and Statistical Manual of Mental Disorders
ED: Eating Disorder
EDI-2: Eating Disorder Inventory, 2nd revision
OSFED: Other Specified Eating and Feeding Disorder
RAB: Rating of Anorexia and Bulimia
SASB: Structural Analysis of Social Behavior
SCL-90: Symptom Check List-90

## Footnotes

1 “In order to test for effects of specific SCL-90 subscales, we also tested Depression and Obsession-compulsion as separate variables since these were deemed most relevant to Self-blame and long-term outcome in AN, respectively. However, no baseline differences between outcome groups were found; *p*s .973 and .964.”
